# Assessing Cat Welfare: A Literature Review on Behavioural, Physiological and Health Parameters with a Focus on Animal-Assisted Services (AAS)

**DOI:** 10.3390/vetsci13060581

**Published:** 2026-06-13

**Authors:** Giulia Russo, Carmen Borrelli, Karen L. Overall, Chiara Mariti

**Affiliations:** 1 Department of Veterinary Sciences, University of Pisa, 56124 Pisa, Italy; giulia.russo@phd.unipi.it (G.R.); carmen.borrelli@phd.unipi.it (C.B.); 2 Department of Biomedical Sciences, Atlantic Veterinary College, University of Prince Edward Island, 550 University Ave, Charlottetown, PE C1A 4P3, Canada; koverall@upei.ca

**Keywords:** cat, feline, animal-assisted services, animal-assisted intervention, welfare assessment, health, behaviour, physiology

## Abstract

Cat welfare assessment in Animal-assisted aervices (AAS) is necessary to ensure engagement in AAS does not impair welfare. An initial systematic search conducted according to Preferred Reporting Items for Systematic Reviews and Meta-Analyses (PRISMA) guidelines revealed a complete lack of studies specifically addressing this topic. Consequently, a second-step search was performed, widened to the existing tools for monitoring cat welfare in general. Three scoping reviews using PRISMA guidelines were performed, focusing on the three principal fields of welfare assessment: health, behaviour, and physiology/endocrinology. Studies published on Scopus in 2011–2026, written in English and assessing feline (*Felis catus*) welfare were selected. A total of 2728 records were identified, of which 43 met the inclusion criteria (including 7 reviews and 1 scientific report); the methods used to assess cat welfare were summarized and discussed for their potential application in AAS. Although multiple tools are available, most are context- or disease-specific and some are not validated. Approximately half of the studies employed more than one indicator or method, with behavioural parameters being the most frequently used through questionnaires or scores. This work provides a practical framework to support veterinarians, handlers, and researchers in the selection of appropriate tools for cat welfare monitoring in AAS.

## Simple Summary

Cats are increasingly involved in animal-assisted services (AAS), making it important to ensure their welfare during these activities. However, using a systematic search, we found that no studies specifically address how to monitor cat welfare in this context. Therefore, we performed a new search to provide an overview of currently available methods for assessing cat welfare across three main fields: health, behaviour, and physiology. The results are discussed to identify which methods are more suitable for inclusion in a multiparameter protocol for monitoring cat welfare in AAS. This review should help veterinarians, practitioners, handlers, and researchers engaged in AAS to monitor the welfare of the involved cats.

## 1. Introduction

Animal-assisted interventions (AAIs), or animal-assisted services (AAS) [1], are structured interactions between humans and animals with therapeutic, rehabilitative, educational, and recreational aims [2]. These interventions involve the engagement of domestic animals to support and enhance the health and well-being of individuals affected by physical, neuromotor, mental, and psychological disorders [3].

According to their objectives, delivery methods, and the professionals involved, AAS can be classified into animal-assisted treatment (AATx), animal-assisted education (AAE), and animal-assisted support programmes (AASPs), based on specific characteristics of the project [1].

At the international level, several organizations (e.g., the International Association of Human–Animal Interaction Organizations—IAHAIO, Pet Partners, the Association of Animal-Assisted Intervention Professionals—AAAIP) provide guidelines, training, and standards for professionals operating in AAS contexts. In Italy, AAS are strictly regulated by national guidelines directly developed by the Ministry of Health [3], which has defined the type of domestic animals that can be involved in AAS. The most common animals involved are dogs and horses, although donkeys, cats, and rabbits are also included in the guidelines [4]. The animal’s suitability for participation is determined through specific health and behavioural assessments, and each animal is certified by a veterinarian with specialized training in AAS [3,5]. Each animal must be in good health at the time of involvement in AAS project, and before each session. Only adult animals are eligible for participation, and certain physiological conditions—such as oestrus, pregnancy, or lactation—temporarily preclude involvement. In addition to the strict health criteria, behavioural suitability plays a crucial role. Animals must exhibit stable and appropriate behaviour, including sociability, adaptability, and a willingness to interact with humans and other animals, both independently and when under guidance. When necessary, animals may undergo targeted training to enhance cooperation with handlers and interest in engaging in activities. All health and behavioural assessments, and monitoring data must be systematically recorded in the animal’s clinical documentation.

In fact, monitoring the welfare of animals involved is crucial due to the potentially stressful stimuli and demanding situations they may face [6]. Stimuli vary depending on the setting in which AAS occur (e.g., kindergartens, hospitals, prisons, and nursing homes) [7], duration of transport to the facility [7], the type of AAS, the quality of the relationship with the handler [5], and factors such as the animal’s individual characteristics [8].

In recent years, AAS have gained increasing attention across various settings and have been recognized as innovative approaches that can improve health and well-being in children, older adults, individuals with disabilities, and people living in institutional contexts. However, little is known about good practices to safeguard welfare of animals involved, especially with respect to less frequently involved species, like cats. Cats often experience discomfort with transportation and environmental changes, such as visiting unfamiliar places [9]. Changes in the familiar environment of cats and exposure to unfamiliar people and settings, novel stimuli, and loud sounds have also shown to potentially be significant stressors for cats [8,9,10,11]. While there is research on how to monitor welfare, behavioural, and physical conditions of dogs engaging in AAS [7,12,13], such data and literature seem to be lacking for cats.

Accordingly, the twofold aim of this scoping review paper was to: (a) systematically review and assess the literature regarding monitoring of cat welfare with different methods; and (b) critically select the most suitable ones to be applied during AAS.

## 2. Materials and Methods

The present review was structured in two steps. In Step 1, a systematic review following PRISMA (Preferred Reporting Items for Systematic reviews and Meta-Analyses) guidelines [14] was conducted to investigate welfare assessment methods for cats involved in AAS, with the aim of evaluating the quality of available evidence and identifying gaps in current methods. As no specific studies on cat welfare assessment in AAS were identified, Step 2 focused on welfare assessment methods described for cats in general, by performing three scoping reviews (PRISMA-ScR) [15] in three fields (health, behaviour, and physiology). Step 2 aimed to address gaps in the literature by mapping the available studies across the three fields.

### 2.1. Step 1

#### 2.1.1. Rationale

Cat welfare assessment in AAS is necessary as cats become more frequently engaged in AAS across settings. Feline ethology is unique and such assessments are needed to decrease the likelihood of welfare impairment.

#### 2.1.2. Objectives

The objective of the study was to systematically identify and evaluate monitoring protocols, assessment methods, and indicators used to assess the welfare of cats involved in AAS, according to Preferred Reporting Items for Systematic reviews and Meta-Analyses (PRISMA) 2020 checklist [14].

#### 2.1.3. Eligibility Criteria

Inclusion criteria: Studies on domestic cats (*Felis catus*) that describe, use or validate monitoring protocols, assessment tools or behavioural, physiological, health indicators of cat welfare during AAS.

All types of peer-reviewed publications were included (e.g., original research articles and systematic, scoping, and narrative reviews); books, notes, letters, and grey literature were also considered. There were no limitations based on years of publication.

Exclusion criteria: Studies focused on human well-being without reporting any indicators, observations or assessment of the cat’s welfare; publications not in English; publications without an available full text.

#### 2.1.4. Information Sources and Search Strategy

The online databases Scopus and PubMed were used to conduct the searches (last date of search 16 January 2026). Boolean search phrases (see below) were used to retrieve the relevant literature. Keywords were used in various combinations with Boolean operators “OR” and “AND” used to combine search terms:

(cat OR cats OR feline OR felines OR “domestic cat” OR “*Felis catus*”) AND (welfare OR “well-being” OR wellbeing) AND (“animal assisted intervention*” OR “animal assisted service*” OR “animal assisted therap*” OR “animal assisted education*” OR “animal assisted activit*” OR “therapy animal*” OR “cat therapy” OR “feline assisted intervention*”).

#### 2.1.5. Selection Process

All searches were exported from Scopus into Excel and duplicates were removed manually. Study selection was carried out in two rounds by a reviewer (GR). The first round included title and abstract screening considering the eligibility criteria and searches were classified as excluded (not about cats or not in English), or included (i.e., reports sought for retrieval).

The second round consisted of the full-text screening of included studies, to ensure the study was relevant to the research objectives.

### 2.2. Step 2

#### 2.2.1. Rationale

The literature on cat welfare assessment in AAS is lacking. Selecting a welfare monitoring protocol applicable in AAS with cats is necessary and should use tools described for the assessment of cat welfare in general.

#### 2.2.2. Objectives

The objective of the study is to obtain an overview of existing tools to monitor cat welfare in general, by performing three separated scoping reviews (following PRISMA extension for scoping reviews—PRISMA-ScR) [15] in the three principal fields of welfare assessment: health, behaviour, and physiology/endocrinology.

The ultimate and practical aim of this work is to guide the selection of a multiparametric protocol for assessing cat welfare to be applied for AAS.

#### 2.2.3. Eligibility Criteria

Inclusion criteria: We included studies on domestic cats (*Felis catus*) that focused on the development, validation or practical application of methods or indicators for assessing cat welfare in various contexts (e.g., shelters, veterinary clinic), applicable also in AAS. Publications were included if they were published over the past 15 years (i.e., between 2011 and 2026, up to the last search date) and were written in English. There were no limitations for inclusion of studies: all types of peer-reviewed publications were included (e.g., original research articles and systematic, scoping, and narrative reviews), and books, notes, letters, and grey literature were also considered in the database.

Exclusion criteria: We did not include papers not focusing on the feline species (e.g., human, dog, cattle), addressing other topics (e.g., diseases, treatments, therapies with drugs, vaccination, zoonoses, urban policies) or not proposing a method or indicator for assessing cat welfare applicable in AAS. Considering the methods applicable in AAS, publications were excluded following the criteria below:

• Invasive methods: Tools or methods that would substantially increase animal distress (e.g., physical restraint of the cat, invasive sampling techniques such as blood sampling, or the use of cumbersome wearable devices that may impair natural behaviour).
• Interfering procedures: Methods that would disturb, interrupt or interfere with the AAS activities (e.g., procedures requiring prolonged separation of the cat from AAS participants, or assessments that require stopping the session).
• Methods that cannot be easily applied in the field: Methods, tools, or parameters that require specific, expensive, and complex equipment that is difficult to transport and would not permit quick assessment (e.g., special laboratory-based analyses, imaging technologies not adaptable to field conditions, multi-step protocols requiring long preparation time).

When possible, these criteria were applied during title and abstract screening, and then through the full-text round.

Publications were not included if they were published before 2011, not written in English or if the full text was not available.

#### 2.2.4. Information Sources and Search Strategy

The online database Scopus was used to conduct the three separate searches (last date of search 3 February 2026). Boolean search phrases (see below) were used to retrieve the relevant literature, ranging from 2011 to 2026, in English. The literature search string was identical for all three fields, with the specific addition based on the field (health, behaviour, and physiology). Keywords were used in various combinations with Boolean operators “OR” and “AND” used to combine search terms:

- Health: (cat OR cats OR feline* OR “domestic cat*” OR “*Felis catus*”) AND (assess* OR evaluat* OR monitor* OR measur* OR indicator* OR paramet* OR biomarker*) AND (welfare OR wellbeing OR well-being OR “quality of life”) AND (health).
- Behaviour: (cat OR cats OR feline* OR “domestic cat*” OR “*Felis catus*”) AND (assess* OR evaluat* OR monitor* OR measur* OR indicator* OR paramet* OR biomarker*) AND (welfare OR wellbeing OR well-being OR “quality of life”) AND (behavior* OR behaviour*).
- Physiology: (cat OR cats OR feline* OR “domestic cat*” OR “*Felis catus*”) AND (assess* OR evaluat* OR monitor* OR measur* OR indicator* OR paramet* OR biomarker*) AND (welfare OR wellbeing OR well-being OR “quality of life”) AND (physiolog* OR endocrin* OR hormon*).

The search for the three scoping reviews was initially performed also on PubMed. With the same strings (and filters) reported above for Scopus, a very high number of articles was obtained (over 6000 for each search). However, the authors noticed some issues: for instance, very recent and related articles were not included; many articles were unrelated but appeared in the list because the term “CAT” was used for catalases. In order to solve the latter, authors attempted to add “NOT catalase” or “NOT enzym*” to the search string, but it did not work because it also removed articles that could have met the inclusion criteria. For these reasons, we decided not to use PubMed and instead to continue with the PRISMA method in Scopus (500–1600 results in each field).

#### 2.2.5. Selection Process

All searches were exported from Scopus into Excel in three different sheets. Before the first round of selection process, the two reviewers (GR and CB) established eligibility criteria which were applied independently during the screening process of titles/abstracts. Searches were classified as “excluded” or “provisionally included”. At this point, included studies were exported into one Excel sheet to automatically remove duplicates. Publications belonging to more than one field (e.g., both in “behaviour” and “physiology”) were placed into an additional Excel table so that the references would not be lost once the duplicates were removed. After excluding records that were not retrieved (i.e., full text not found), a second screening round was conducted. This round involved a full-text review, after which studies meeting the eligibility criteria were included in the analysis. Due to the large number of studies selected for full-text assessment, the second screening round was primarily conducted by one reviewer (GR). A second reviewer (CM or CB) was consulted when the primary reviewer was unsure about classification and their decision was used.

#### 2.2.6. Data Item and Synthesis of Results

Data items extracted from each study included: (i) study characteristics and general information (e.g., author, year, doi); (ii) welfare indicators and assessment methods (e.g., physiological and/or behavioural parameters used), with related descriptions (e.g., questionnaires, scoring systems); (iii) methodological features (e.g., validation of the methodology and who conducted the assessment); and (iv) applicability within the feline AAS context. Considering that the objective was to map assessment methods and indicators, studies were grouped according to the types of assessment tools used. This approach facilitated a comparative analysis of the applicability of those tools within real-world feline AAS.

## 3. Results

### 3.1. Step 1

**Figure 1:**
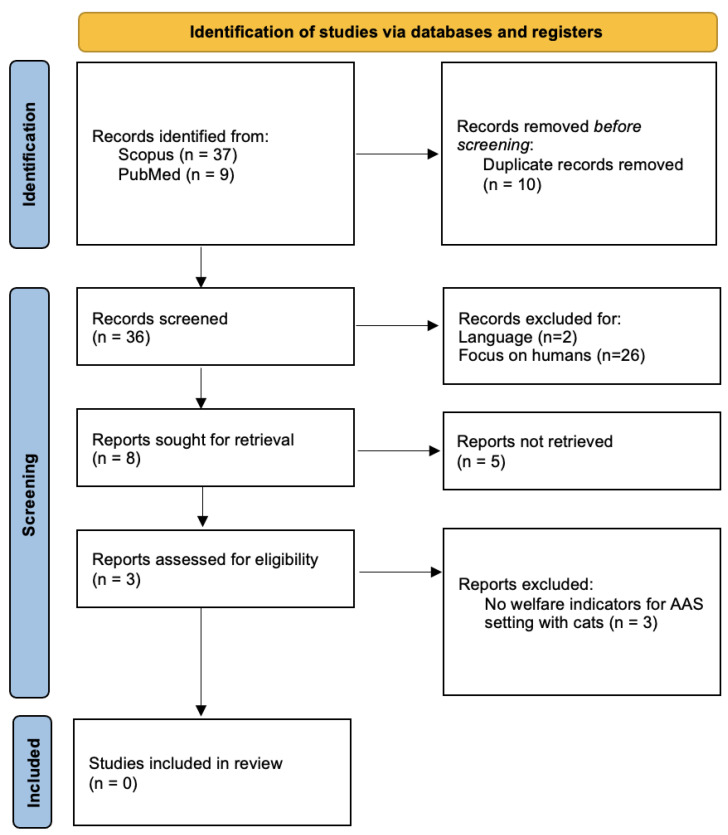
Flow diagram of Step 1 of the study.

A total of 46 studies were identified and screened as reported in Figure 1. Ten duplicates were removed; 36 records were screened by title and abstract considering eligibility criteria, of which 28 were excluded for language (*n* = 2) or focus on humans (*n* = 26); the remaining eight were screened and all discarded because they were not available in full text (*n* = 5) or did not assess animal welfare in AAS (*n* = 3).

### 3.2. Step 2

**Figure 2:**
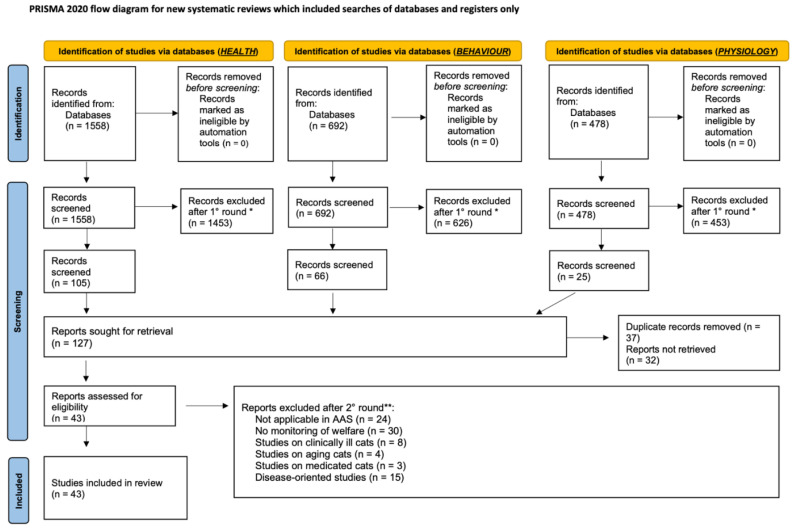
Flow diagram of the Step 2 of the study. (*) First round of the selection process: screening by titles/abstracts. (**) Second round of the selection process: screening by full text.

A total of 2728 articles (see Figure 2) were identified: 1558 for health, 692 for behaviour and 478 for physiology. All were initially screened by title/abstract, and 2532 articles (1453 for health, 626 for behaviour and 453 for physiology) were excluded. At this point, a total of 196 studies (105 for health, 66 for behaviour and 25 for physiology) remained and, after removing duplicates (*n* = 37) and records not retrieved (*n* = 32), 127 studies were screened by full text. Reports were excluded at the full-text screening (second round) for the following reasons:

• Not applicable to AAS (*n* = 24): The proposed methods or indicators, although potentially relevant for animal welfare assessment, were not feasible in the AAS context.
• No welfare monitoring (*n* = 30): The studies did not report results or provide evidence relevant to the monitoring or evaluation of animal welfare.
• Studies on clinically ill cats (*n* = 8): The studies focused mainly on clinically ill animals, which may influence the assessment of welfare indicators and limit applicability to healthy cats. If studies included a group of ill cats but the tool was also applicable to healthy cats (e.g., already validated and generalizable), they were kept in the analysis.
• Studies on ageing cats (*n* = 4): The studies focused on ageing cats, using indicators specific to this life stage and associated conditions.
• Studies on medicated cats (*n* = 3): The studies focused mainly on animals undergoing pharmacological treatments (e.g., anesthesia, palliative care) which may influence physiological and behavioural welfare indicators. If studies included treated cats to develop a tool applicable to healthy cats, they were kept in the analysis.
• Disease-oriented studies (*n* = 15): The studies were specifically designed to investigate diseases (e.g., questions specific to symptoms) and were not generalizable to overall welfare assessment.

#### Synthesis of Results

A total of 43 studies were assessed for eligibility and are reported in Tables S1 and S2 (see Supplementary Materials). Of the 43 eligible studies, 7 were reviews [10,16,17,18,19,20,21], and 1 was a scientific report [22]. Among the research articles (*n* = 36), 20 assessed cat welfare using more than one parameter (e.g., Psychological Quality of Life, PQOL, and Cat Stress Score, CSS) and, among these, 9 included parameters belonging to different categories (e.g., both physiological and behavioural). Overall, 30 studies used behavioural parameters, 10 used physiological parameters, and 7 used health parameters. In addition, 31 studies used at least one validated parameter, and 15 articles used at least one parameter based on perception of owners (e.g., questionnaires). The most frequently used welfare method was the Cat Stress Score (CSS) (*n* = 9), a seven-level (1 = relaxed, 7 = terrified) scoring system based on several cat attributes (body, belly, tail, ears, head position) (see Supplementary Materials, Table S3).

## 4. Discussion

To the authors’ knowledge, and in light of the results of Step 1, this review represents the first attempt to identify monitoring protocols for assessing cat welfare within the context of AAS. This scoping review became necessary to provide an overview of the existing methods and indicators that may be applicable in this field. Forty-three records were identified, selecting those with potential applicability to AAS.

The AAS monitoring protocol for animal welfare should be evaluated considering the potential effects of the session(s) in terms of intensity and duration, and through the collaboration between the veterinarian and the handler. Welfare monitoring must be conducted in a non-invasive and minimally disturbing manner, whenever possible. The use of invasive or cumbersome wearable devices, performing blood sampling or clinical procedures requiring physical restraint of the cat are all examples of potentially valid clinical assessments for other purposes, but not appropriate in this context. Cats are sensitive to handling procedures [17], compared with many other species, which may especially affect their behaviours during demanding activities. Instead, emphasis should be placed on hands-off, non-invasive welfare monitoring approaches, without interfering with the cat’s behaviour and comfort, or with the human–animal interaction itself.

Welfare assessment in animals involved in AAS can be divided in three temporal dimensions: short-, medium- and long-term evaluation. Different methods and/or indicators may be more appropriate for conducting assessments that differ temporally.

In a “short-term evaluation”, the assessment may be conducted before, during, and after AAS. In this context, the veterinarian and the handler evaluate the animal’s current condition, including cat state prior to the start of the activity, real-time monitoring during the session, and the short-term effects of AAS on the animal.

It is crucial to conduct a pre-AAS assessment before the start of an AAS session to ensure that the cat is healthy and free from ongoing pain. Various pain assessment scoring systems are available in the literature, including the Feline Grimace Scale (FGS) [21,23,24,25], the Glasgow Composite Measure Pain Scale—Feline (Glasgow CMPS-Feline) [21,24,26], the UNESP-Botucatu Multidimensional Feline Pain Assessment Scale Short Form (UFEPS-SF) [21,24,27], and the Colorado State University Feline Acute Pain Scale (CSU-FAPS) [28,29]. These validated scales assess acute pain in cats [21,24].

In particular, the Glasgow CMPS-Feline and UFEPS-SF are easy to use and relatively fast, but they involve handling the cat, restricting their use to pre- and post-AAS assessments by the veterinarian or handler.

The FGS is composed of a visual scale with drawings and so could be quickly used as a pain monitoring assessment pre- or post-session, analyzing the facial expressions of the cat without handling, and so is preferable as to not interfere with pre-AAS session assessment and to not artefactually add stressful stimuli when used as a post-session assessment.

The CSU-FAPS has been preliminarily validated [29] and, as for FGS, is a scale based also on visual representations that allows relatively rapidly scoring before and after AAS [28]. Drawings and simple descriptions help the observer more easily score the behaviour and improve understanding of behavioural patterns. The CSU-FAPS also allows potential areas of pain to be noted and marked on a body-area schematic. Cats with an ongoing painful condition should not be involved in AAS, so pain assessment could be important also in the suitability assessment. Such assessments may also help to identify more obscure pain signals which would help to exclude ineligible cats. The best utility of such scales is to ensure the absence of pain conditions at the start of the AAS session, and to confirm that no pain is due to AAS (e.g., accidental injury) after the session. Health parameters can be easily assessed during the pre-AAS evaluation to exclude animals with current illness. Vojtkovská et al. [30] and Zito et al. [31] used a visual tool to assess the general health condition of cats using body condition score (BCS), coat condition, eye, ear and nose discharge, respiratory sounds, abnormal posture, and injury in a non-invasive and rapid manner.

In general, pre- and post-AAS assessments are characterized by the collection of parameters that can be compared across time points (e.g., T0 before and T1 after the AAS session). Ideally, an additional baseline should also be included which includes measurements collected prior to transport on the day of the session and/or on a separate day without AAS activities. These types of repeated evaluations are most commonly based on physiological parameters. In this review, only 2/10 articles assessed a physiological parameter as the only indicator [32,33]. In the remaining eight, the physiological assessment was associated with other types of indicators (e.g., behavioural and physiological parameters in the assessment protocol).

Salivary cortisol has been commonly used as an indicator of acute stress [34], but there are concerns and limitations about the collecting procedure in cats [35,36] that may result in insufficient samples for statistical analysis [32,35]. In one study, only a few samples yielded enough saliva for analysis and the salivary cortisol did not correlate with plasma cortisol levels [36]. Owing to these important methodological issues, the authors discourage salivary sampling for acute physiological assessment in cats and other species (e.g., rabbits) during AAS, with preference given to less invasive matrices, proposed below. Other common physiological parameters used in welfare assessment protocols are blood pressure, heart rate, heart rate variability (HRV), respiratory rate, and rectal or body temperature. In cats, handling and restraint can significantly affect these indicators, potentially influencing their interpretation [17,37]. Respiratory rate [35] is a non-invasive parameter that can be evaluated before and after AAS sessions, assuming the sessions are done in temperature-controlled environments. Respiratory rate may reflect stressful conditions, if season and environmental temperature are not influencing factors. Moreover, the interpretation of physiological stress-related parameters is complex, and the absence of changes or variations in these indicators does not necessarily indicate good or poor welfare [38].

Csiplo and Popescu [39] assessed owners’ observations during veterinary visits, through a questionnaire based on their familiarity with their pets’ typical behaviour. This tool could be used to assess stress before, during and after an AAS session by the handler. Considering that in [39] perceived stress levels peaked immediately after entering the clinic and declined after approximately 10 min, identifying the most stressful time points for cats also in the AAS context may improve management strategies. However, a questionnaire focused on owners’ perception of stress in cats [40] found that owners are not always able to recognize stress in their cats. This is a crucial point, considering that the handler is the first person responsible for guaranteeing the cat’s welfare during AAS. For owners, travelling is one of the most stressful situations [40]. Existing guidance on transporting cats and introducing them to new environments may also apply to novel AAS settings (e.g., first-time facility visits) [10].

The real-time monitoring during the activity must be easy to complete, immediate, and fast. These characteristics reflect the most utilized method in the publications included in the current review [35,41,42,43,44,45,46,47,48]: the Cat Stress Score [49]. With the CSS, the observer assigns a seven-level (1 = relaxed, 7 = terrified) score to each attribute (body, belly, tail, ears, head position) and then calculates the mean. The CSS may represent a good tool to assess whether a cat, during an AAS session, is stressed and at what intensity. Nevertheless, some authors have criticized the CSS for lack of correlation with urinary and fecal cortisol [50,51]. This discrepancy likely reflects the interpretation of cortisol as a unitary measure, despite its complex dynamics, and it may be explained by differences in the assessment periods. The CSS reflects short-term changes, representing an instantaneous measure of the cat’s communicated perception of the response to the situation at that time, whereas fecal and urinary cortisol are more indicative of medium-term changes reflecting physiological response over longer time periods and suggesting that additional factors and interactions may also contribute. The use of a scale with a scoring system like the CSS allows the observer to perform an assessment without touching or handling the animal, and without interfering with the AAS activity.

Additionally, using a scale to rate behaviours can be a useful way to summarize observations and ensure that a common language is used [52]. A study included in this review utilized a simplified and validated version of the CSS, the Simplified Feline Stress Scale (SFSS), with three levels (1 = low, 3 = marked stress), applied by a trained observer before, during, and after the measurement of rectal temperature. Cats with moderate to marked signs of stress exhibited higher rectal temperatures compared to those with lower SFSS stress levels. The range of temperature changes (low stress 36.2–39.9 °C; marked stress 37.3–40.0 °C) indicates that this response represents a physiological response to a stressor, and not somatic illness [41]. Rectal temperature measurement itself is an invasive and potentially stress-inducing procedure [53]; therefore, a behavioural evaluation might represent a valuable proxy [41]. The SFSS can be used as an alternative to the CSS in future studies [41], as it includes fewer scoring levels than CSS (1–3 vs. 1–7), potentially facilitating its application by handlers who are already performing multiple functions during an AAS session.

The Fear Anxiety Stress (FAS, Fear Free^®^) scoring system proposes a score based on body language. It is easy to use and allows immediate evaluation through visual representation; for this reason, it can be repeated during the session (every 5 or 10 min for example), representing a snapshot of the cat’s level of well-being and the direction of any changes over the time period assessed. FAS scoring might be associated with other tools. For instance, Ellis [52] used an adapted version of the FAS system together with three additional scales ranging from 0 to 5: Food Intake Summary (FIS) score, Participation in Play (PIP) score, and Response to Petting (RTP) score [52]. The PIP and RTP may be easily applied when assessing the cat’s response to human interaction (petting and playing) during the session, while the FIS, based on the last two meals, could be useful for assessing the general well-being and health status of the cat before the start of a session, as an indicator associated with stress [54]. The FIS could be adapted for AAS by offering food to the cat and assessing the response, as this approach is likely to be effective only when the animal is not already in a heighted state as a response to a prior stressor [55].

Video analysis of AAS sessions may be used as a tool for subsequent behavioural observations, using a selected ethogram [11,56,57,58] to provide qualitative and/or quantitative descriptions, counts, and ratings of the relevant behaviours (e.g., social interaction, signs of stress or pain, feline emotions, etc.) and to validate the use of the other scales in the particular AAS application. Video recording should be conducted without interrupting or disturbing the session itself, to not alter the cat’s behaviour and/or interfere with the animal’s motivation to participate in AAS.

The second assessment, the “medium-term evaluation”, can be conducted on the day following the session or within the subsequent week after AAS and may involve both physiological parameters and questionnaires.

Three papers evaluated fecal cortisol or fecal glucocorticoid metabolite concentration [11,42,46] which were determined in association with other measures (e.g., CSS, Feline Temperament Profile (FTP), questionnaires, body weight). Measurement of serum and/or salivary cortisol is not suitable for short-term assessment due to the difficulty of collecting blood and saliva samples and the distress this would cause for cats who are not taught to cooperate with such interventions [59]. Fecal samples are a non-invasive indicator for measuring cortisol in cats, reflecting approximately the past 24 h [60,61], and could be a valid method to assess an earlier AAS session by collecting samples after the session and the following day. This approach may be limited in multi-cat households unless a marker is fed to the cat (e.g., beet juice-stained treats) or in cats with outdoor access, where sample collection cannot be ensured.

Urinary cortisol reflects recent adrenal activity over several hours up to about a day [62]. In a study reported in this review [63], the measurements of urinary cortisol and oxytocin were associated with data about the frequency of owners’ daily interactions with their cats using a questionnaire. The results showed that no factors were associated with cortisol levels, while social interactions with owners influenced the physiological status (i.e., oxytocin levels) of cats [63]. Oxytocin has recently attracted attention as a positive indicator of animal health, welfare, and physiological assessment of cats living in a non-stressful environment [63]. Oxytocin can be measured by collecting blood and saliva, but the amount of biological matrix needed is relatively high, reducing its applicability in most circumstances. The “long-term evaluation” refers to a period of weeks or even months, during and/or after a project. Long-term evaluations should be primarily conducted by the handler, with support from the veterinarian.

Questionnaires appear to be commonly used in studies on cat welfare assessment, and they are usually completed by owners or facility staff. An example of a questionnaire used for long-term evaluation is the validated Fe-BARQ [64,65,66,67,68] which asks owners to describe the frequency of their cat’s behaviour across a variety of circumstances using five-point scales ranging from never (0) to always (4). The Fe-BARQ could also be utilized to monitor the cat’s behaviour over time (in months) to compare any differences that may occur in the long term, related to the fact that a cat might be working in more projects in the same period or to the consequences of particularly long/stressful AAS projects. A possible limitation of this questionnaire, as well as others, is that it has been validated only in English [64], Spanish [67], and Flemish [68].

Other useful tools could be questionnaires assessing cat quality of life (QoL). The Psychological Quality of Life (PQOL) is a short questionnaire that assesses cats’ behaviour, attitude, and activity [43]. The PQOL fits well as a method to monitor AAS projects, because it includes items about the cat’s interest in playing with objects, toys, and people, and the cat’s common resting and relaxing behaviours.

Items included in the VetMetrica^®^ Survey (health-related QoL, HRQoL) could represent a valid tool to assess the cat’s current emotional state, vitality, and comfort [69,70]. This tool used the handler assessment of whether the cat is active, playful, content, or alert, as well as whether it appears uncomfortable or stiff.

The Cat HEalth and Wellbeing (CHEW) questionnaire has been validated for assessing HRQoL in cats, demonstrating that physical, mental, emotional, and social dimensions identified by caregivers are reliable and valid indicators of feline QoL [71].

Finally, another validated feline QoL survey divided the items into behaviours indicative of “healthiness” (e.g., the cat is seeking attention, being happy and affectionate towards owner) and clinical signs (e.g., the cat has been inactive, ill, has lost weight), allowing changes to be tracked over a long time [72]. This tool could be used on a regular basis, for example every six months, or according to the duration of the single AAS project.

Owner-completed questionnaires have also been used to assess cat behaviour and behavioural problems. The CABIAS is validated and uses a scoring system combining aspects of the frequency (0–7 point scale) and intensity (1–10 visual analogue scale VAS) of the behavioural problem [73]. Although cats involved in AAS should not present behavioural problems, this tool can be administered weekly to assess changes in behavioural parameters as part of a feline welfare assessment [74].

Health-related indicators, like weight, could be valuable in long-term AAS evaluation to monitor overall health status and potential adverse effects associated with excessive workload. Five included studies utilized body weight or body condition score (BCS) as parameters [30,31,42,47,75]. In cats weight loss may indicate impaired welfare, and other already mentioned health-related indicators [30,31] may further reflect possible costs of workload.

Physiological parameters, especially when combined with other methods, may also be helpful in long-term assessments. For example, in this review, when hair cortisol concentration (HCC) was used in assessments, its use was coupled to a validated questionnaire [76], or to a questionnaire and nail cortisol concentration (NCC) [77]. HCC has emerged as a valuable, non-invasive biomarker for monitoring long-term cortisol levels in animals [60,78] as it measures HPA activity over weeks and months [79]. However, HCC can be influenced by many factors, including but not limited to chronic stress levels [80]. Notably, a recent study has proposed a novel, low-stress method for hair sampling in cats, suggesting that combing may represent a practical and cat-friendly alternative for cortisol quantification [81]. By avoiding repeated shave–reshave sampling, owner compliance may be improved, and repeatability can be better assessed [81], supporting its potential use for assessing chronic stress over extended periods (long-term evaluation) in the context of AAS. In general, when interpreting possible changes over time (long term evaluation), it is important to consider that behavioural, physiological, and health-related variations may also be influenced by changes in the cats’ environment, general health status, or other concurrent factors and may not be exclusively related to the AAS project. However, the reported changes need to be carefully considered in order to evaluate whether the cat is experiencing stressful conditions that may make it unsuitable for working in AAS, at least temporarily.

The choice of which of these tools (see Supplementary Materials) to use will depend on several factors including the characteristics of the AAS, such as frequency and type of activities, available resources (including financial ones), individual characteristics of the cats, and handler compliance.

This review has some limitations. One is that full-text screening was performed by a single reviewer unless there was uncertainty. In addition, the grey literature was not systematically and separately searched, so unpublished studies may not have been identified. Manual reference screening was performed to support the Discussion section; however, it was not systematically applied to identifying additional studies for inclusion in the review. Most importantly, after cross-checking the literature with which the authors were already familiar, they recognized that the search string may have introduced some biases due to the presence of terms such as assess* and monitor*, which, although broad, may have excluded relevant studies. Although the exclusion of PubMed during Step 2 was done to reduce the inclusion of irrelevant records, this choice may have limited the comprehensiveness of the review and introduced a potential selection bias. Future work on this topic should consider implementing more refined search strategies including PubMed (and other databases, e.g., Web of Science), such as MeSH terms and title-restricted searches.

Some references were added in the Discussion section that were not included in the review itself. A refinement of the search strategy should be considered in future analyses, and separating the three scoping reviews into three systematic reviews would likely provide more accurate insights into the three fields, offering clearer aid for veterinarians and handlers aiming to develop protocols for assessing cat welfare in AAS. Future work will focus on establishing practical guidelines for cat welfare assessment in AAS based on both existing literature (the focus of this review) and possibly new tools.

## 5. Conclusions

In recent years, AAS involving cats have gained increasing attention [68,82]. However, little is still known about how to monitor and ensure the welfare of these cats. This review shows that various methods and indicators are available, but only few are applicable to AAS. The most suitable approaches are behavioural ones, implemented through scoring systems or questionnaires completed by owners/handlers. Additionally, incorporating physiological and health-related parameters into welfare assessments could provide valuable information, provided they are collected in a non-invasive manner and with minimal handling. Indeed, developing a multiparametric protocol applied at different time points (short-, medium-, long-term) would allow for a more comprehensive evaluation of the welfare and quality of life of cats involved in AAS.

## Data Availability

The original contributions presented in this study are included in the Supplementary Materials. Moreover, the review’s materials, protocol and databases are available on the Open Science Framework (OSF) at the following link: https://osf.io/dzmnh/overview?view_only=ec0bbfebdfa440e28e28cd36351c6865 (accessed on 1 June 2026).

## Supplementary Materials

The following supporting information can be downloaded at https://www.mdpi.com/article/10.3390/vetsci13060581/s1. Table S1. Characteristics of eligible articles included in the review (n = 36), with a focus on welfare indicators and assessment methods, measurement approaches, study contexts, validation status of the methods, and applicability in feline Animal-Assisted Services (AAS). When the same indicator is reported across multiple studies, its complete description is provided only once (e.g., Cat Stress Score, Fe-BARQ). Abbreviations: BCS, body condition score; CSS, Cat Stress Score; ECG, electrocardiography; EFSA, European Food Safety Authority; ELISA, enzyme-linked immunosorbent assay; FAS, Fear, Anxiety, and Stress; Fe-BARQ, Feline Behavioural Assessment and Research Questionnaire; FGM, fecal glucocorticoid metabolites; FGS, Feline Grimace Scale; FIS, Food Intake Summary score; FTP, Feline Temperament Profile; Glasgow CMPS-Feline, Glasgow Composite Measure Pain Scale—Feline; HCC, hair cortisol concentration; HRQoL, health-related quality of life; HRV, heart rate variability; IPAQ, International Physical Activity Questionnaire; MFS, Motor Fitness Scale; NCC, nail cortisol concentration; PIP, Participation in Play score; PQOL, Psychological Quality of Life; QoL, quality of life; RR, respiratory rate; RTP, Response to Petting score; SFSS: Simplified Feline Stress Scale; UFEPS-SF, University of Melbourne Feline Pain Scale—Short Form; WHO-5, World Health Organization—Five Well-Being Index. Table S2. Characteristics of eligible included reviews (n = 7) and scientific report (n = 1), with a focus on welfare indicators and assessment methods, measurement approaches, study contexts, validation status of the methods. Abbreviations: EFSA, European Food Safety Authority; FGS, Feline Grimace Scale; Glasgow CMPS-Feline, Glasgow Composite Measure Pain Scale—Feline; QoL, quality of life; UFEPS-SF, University of Melbourne Feline Pain Scale—Short Form. Table S3. Synthesis of results organized into category, subcategory, number of studies in each of them (N), and indication of the references for each subcategory.
