# Supplementary material for: Assessing Cat Welfare: A Literature Review on Behavioural, Physiological and Health Parameters with a Focus on Animal-Assisted Services (AAS)
Source: Vet Sci. 2026 Jun 13;13(6):581. doi: 10.3390/vetsci13060581 (PMC13307602; doi:10.3390/vetsci13060581)
Supplement: Supplementary file 1 [file vetsci-13-00581-s001.zip › vetsci-4330177-supplementary.pdf]

Supplementary Materials

Table S1. Characteristics of eligible articles included in the review (n=36), with a focus on welfare indicators and assessment methods, measurement approaches, study contexts, validation status of the methods, and applicability in feline Animal-Assisted Services (AAS). When the same indicator is reported across multiple studies, its complete description is provided only once (e.g., Cat Stress Score, Fe-BARQ). **Abbreviations: BCS**, body condition score; **CSS**, Cat Stress Score; **ECG**, electrocardiography; **EFSA**, European Food Safety Authority; **ELISA**, enzyme-linked immunosorbent assay; **FAS**, Fear, Anxiety, and Stress; **Fe-BARQ**, Feline Behavioural Assessment and Research Questionnaire; **FGM**, faecal glucocorticoid metabolites; **FGS**, Feline Grimace Scale; **FIS**, Food Intake Summary score; **FTP**, Feline Temperament Profile; **Glasgow CMPS-Feline**, Glasgow Composite Measure Pain Scale – Feline; **HCC**, hair cortisol concentration; **HRQoL**, health-related quality of life; **HRV**, heart rate variability; **IPAQ**, International Physical Activity Questionnaire; **MFS**, Motor Fitness Scale; **NCC**, nail cortisol concentration; **PIP**, Participation in Play score; **PQOL**, Psychological Quality of Life; **QoL**, quality of life; **RR**, Respiratory rate; **RTP**, Response to Petting score; **SFSS**: Simplified Feline Stress Scale; **UFEPS-SF**, University of Melbourne Feline Pain Scale–Short Form; **WHO-5**, World Health Organization-Five Well-Being Index.

Reference	Welfare Indicators and Assessment Methods	Measurement type (and Respondent/Observer, if reported)	Study context	Validation status of method/indicator used
Lamon et al., 2026	• Psychological Quality of Life (PQOL)<br>• Adapted Cat Stress Score (CSS)	• PQOL: 11-question assessment of a cat's behaviour, attitude, and activity for shelter cats, completed by trained observer<br>• CSS: non-invasive assessment tool (scoring system) to determine stress levels for shelter cats, performed by trained observer	Shelter	Yes
Bigras-Fontaine et al., 2025	• Cat Stress Score (CSS)<br>• Aggression scale<br>• Simplified Feline Stress Scale (SFSS)	• CSS: performed by trained observer<br>• Aggression scale: scoring system performed by trained observer<br>• SFSS simplified version of the CSS with three levels (1= low stress; 2= moderate stress; 3=marked stress), performed by trained observer	Routine veterinary visit	Yes
Csiplo & Popescu, 2025	• Questionnaire assessing their pets' behavioural responses at different stages of the consultation	• Questionnaire completed by owner	Veterinary visit	No
Lamon et al., 2025	• Hair cortisol concentration (HCC)	• Physiological indicator (hair samples collected by care staff during brushing) using ELISA	Shelter and university	Yes
Lorbach et al., 2025	• HRQoL tool (VetMetrica®)<br>• Clinical data	• Questionnaire completed by owner<br>• Clinical parameters collected included: anemia, muscle condition score, BCS, appetite, continuous variables collected	Within 1 week from veterinary visit	Yes, already validated

		(patient age, body weight, systolic blood pressure, etc.) and others		
Siguín et al., 2025	• 20-Factors Framework, evaluating biosensing wearables for continuous physiological monitoring in animals	• Physiological/health/behavioural parameters (ECG, heart rate, HRV, respiration, temperature, behaviour) and related measurements methods<br>• Case study on thermal stress monitoring in cats (RR and surface temperature)	No	Yes
Marangoni & Steagall, 2024	• Video-based compilation of acute pain behaviours in cats (for veterinary health professionals and owners)	• Behavioural parameters (validated ethogram with video)	No	Yes, already validated
Kogan et al., 2024	• Questionnaire with modified version of the Colorado State University Feline Acute Pain Scale (CSU-FAPS)	• Questionnaire and scoring system, completed by owners	No	Preliminary validation of CSU-FAPS
Chen et al., 2024	• Hair cortisol concentration (HCC)<br>• Questionnaire for quality of life (QoL)	• HCC: Physiological indicator (using ELISA) to evaluate chronic stress<br>• QoL: Previously validated questionnaire completed by owner on physical and mental health, activity behaviour and management of the cat	Veterinary visit	Yes, already validated
McPeake et al., 2023	• Cat Behaviour Issues Assessment Scale (CABIAS)	• CABIAS: Questionnaire with a scoring system combining aspects of the frequency and intensity of the problem behaviour, completed by owners	At home, weekly intervals over a 6-week period	Yes
Powell et al., 2023	• Fe-BARQ [41]	• Fe-BARQ: 100-item questionnaire that asks owners to describe the frequency of their cat's behaviour across a variety of circumstances with five-point scales from never (0) to always (4)	No	Yes, Fe-BARQ already validated
Marangoni et al., 2023	• Ethogram of acute pain behaviours in cats	• Behavioural parameters (ten behaviour categories that could be evaluated by duration and/or frequency): exploratory behaviours, activity, posture and body position, affective-emotional states, vocalization, playing, feeding, post-feeding and facial expressions	No	Yes
Monteiro et al., 2023	• Feline Grimace Scale (FGS)	• FGS: Score system for acute pain assessment in cats that uses changes in facial expressions, completed by owners	No	Yes, FGS already validated

Takagi et al., 2023	• Questionnaire with Fe-BARQ [41] and other indicators (survey on owners: WHO-5, IPAQ, MFS)	• All questionnaires, completed by owners	No	Yes, Fe-BARQ already validated
Ellis, 2022	• Fear, Anxiety, and Stress (FAS) adapted<br>• Response to Petting (RTP) score<br>• Participation in Play (PIP) score<br>• Food Intake Summary (FIS) score	• FAS, RTP, PIP, FIS: All scoring systems (0–5 scale) performed by trained shelter staff	Shelters	No, but high degree of interobserver reliability
Steagall et al., 2022	• Overview of feline acute pain assessment tools (UFEPS-SF, Glasgow CMPS-Feline, FGS)<br>• Behaviours suggestive of acute pain in cats	• UFEPS-SF, Glasgow CMPS-Feline, FGS: Scoring systems<br>• Behavioural parameters	Veterinary clinic	Yes, already validated
Nagasawa et al., 2022	• Urinary cortisol and oxytocin concentration<br>• Questionnaire based on previous studies	• Physiological parameters (urine samples for urinary cortisol, oxytocin, and creatinine using ELISA)<br>• Questionnaire on cat housing environment, individual cat variables, frequency of daily interactions with their cats on a 5- and 7-point Likert scale, Feline Five questionnaire on cat personality, completed by owners	No	Yes, Feline Five already validated
Paz et al., 2022	• Respiratory rate and salivary cortisol<br>• Social interaction score<br>• Cat Stress Score (CSS)	• Physiological parameters<br>• Social interaction score and CSS: Scoring systems, completed by an observer	Veterinary clinic	Yes, CSS already validated
Carlisle et al., 2021	• Feline Temperament Profile (FTP)<br>• Faecal cortisol concentration and body weight<br>• Cat Stress Score (CSS)	• FTP: Objective measure of cat temperament with scores compiled from 10 items, performed by trained staff<br>• Physiological and health parameters<br>• CSS: Scoring system, performed by trained staff	Shelter and home	Yes, CSS and FTP already validated
de Assis & Mills, 2021	• Feline welfare assessment tool	• Questionnaire about change in 21 behavioural and health elements, completed by owner	No	Yes
Contreras et al., 2021	• Hair and nail cortisol concentrations (HCC and NCC)<br>• Questionnaire based on previous studies [41,54]	• HCC and NCC: Physiological parameters (hair and nail samples)<br>• Medical history and cat daily lifestyle questionnaire completed by owner	No	Yes

Nicholson & O'Carroll, 2021	• Ethogram/guide for identifying feline emotions	• Behavioural parameters (ethogram)	No	Yes
Vojtkovská et al., 2021	• Assessment protocol of cats' health	• Health parameters 5-point scale (body condition, eye and nose discharge, respiratory sounds, coat condition, and abnormal posture)	Shelter	No
Kong et al., 2021	• Body condition score (BCS), coat condition and signs of oculo-nasal discharge	• Health parameters	Shelter	No
Van Der Leij et al., 2019	• Cat Stress Score (CSS)<br>• Body weight	• CSS: Scoring system performed by a trained observer<br>• Health parameter	Shelter	Yes, CSS already validated
Stella & Croney, 2019	• Faecal glucocorticoid metabolites (FGM) concentration<br>• Ethogram<br>• Questionnaire assessing personality traits and sickness behaviours	• FGM: Physiological parameters (faeces samples)<br>• Behavioural parameter<br>• Questionnaire completed by owner	Veterinary clinic	Yes, for FGM concentration
Zito et al., 2019	• 5-component visual health-related welfare assessment scale	• Health parameters (BCS, coat condition score, nose and eye discharge score, ear crusting score, and injury score)	No	No
da Silva et al., 2017	• Salivary cortisol concentration	• Physiological parameter using ELISA	Shelter	No
Tatlock et al., 2017	• Assessment of feline health and quality of life (QoL)	• Questionnaire completed by the owner	Veterinary clinic	Yes
Mariti et al., 2017	• Assessment of owner perception of cat stress	• 42-item questionnaire adapted from a previous study, completed by owner	No	No
Loberg & Lundmark, 2016	• Cat Stress Score (CSS)<br>• Ethogram on social behaviours	• CSS: scoring system<br>• Behavioural parameters, collected by an observer	helter (indoor)	Yes, CSS already validated
Pereira et al., 2016	• Cat Stress Score (CSS)<br>• Scale of handling<br>• Owner opinion about behaviour of the cat during the consultation	• CSS and Scale of handling: scoring systems performed by an observer<br>• Questions to the owner	Veterinary clinic	Yes, CSS already validated
Freeman et al., 2016	• Cat HEalth and Wellbeing (CHEW) Questionnaire	• Eight domains and 33 items-questionnaire (HRQoL tool)	No	Yes
Rehnberg et al., 2015	• Feline temperament profile (FTP)<br>• Faecal glucocorticoid metabolite (FGM) concentration<br>• Cat Stress Score (CSS)	• FTP: Objective measure of cat temperament, scores compiled from 10 items<br>• FGM: Physiological and health parameters<br>• CSS: Scoring system	No	Yes, CSS and FTP already validated

Vinke et al., 2014	• Cat Stress Score (CSS)	• Scoring system performed by an observer	Shelter	Yes, CSS already validated

Table S2. Characteristics of eligible included reviews (n=7) and scientific report (n=1), with a focus on welfare indicators and assessment methods, measurement approaches, study contexts, validation status of the methods. **Abbreviations: EFSA**, European Food Safety Authority; **FGS**, Feline Grimace Scale; **Glasgow CMPS-Feline**, Glasgow Composite Measure Pain Scale–Feline; **QoL**, Quality of Life; **UFEPS-SF**, University of Melbourne Feline Pain Scale–Short Form.

**Reference**	**Welfare Indicators and Assessment Methods**	**Measurement type (and Respondent/Observer, if reported)**	**Study context**	**Validation status of method/indicator used**
Lamon et al., 2023	• Overview of the published welfare and quality of life assessments that are available for shelter cats	• Validated ethogram-based assessments<br>• Validated physiological assessment<br>• Non-validated welfare assessment methods	Shelter	Yes
Jahn & DePorter, 2023	• Stress management in preparation of an air travel, applicable also in other types of travel	• Physiological and behavioural effects of short- or long-term stress related to travel	Different new environment	No
Candiani et al., 2023	• Protocol developed for assessing welfare of cats and dogs in commercial breeding establishment (EFSA scientific report)	• Assessment protocol with questions on housing, health and painful surgical procedures	Commercial breeding establishment	No
Fulmer et al., 2022	• Overview of QoL assessment tools	• Questions designed for pet owners and veterinarians to measure the QoL of animals	No	Yes (for 5 out of 5 eligible studies for cats)
Vojtkovská et al., 2020	• Overview of assessment of the welfare of cats in shelters	• Behavioural, physiological and health indicators with an application in both practical and scientific contexts	Shelter	Yes
Foreman-Worsley & Farnworth, 2019	• Behavioural and welfare measures in indoor cats	• Behavioural and physiological parameters, questionnaires	Indoor environments	Yes
Steagall & Monteiro, 2019	• Feline acute pain scales: UFEPS-SF, Glasgow CMPS-Feline, Feline Grimace Scale	• All scoring systems	Veterinary clinic	Yes, already validated
Mills et al., 2014	• Guide on stress impact on dogs and cat's health	• Behavioural, physiological and health parameters	No	No

Table S3. Synthesis of results organized in category, subcategory, numbers of studies in each of them (N), indication of the references for each subcategory.

Category	Subcategory	N	References
**Study type**	Total included studies	43	(Bigras-Fontaine et al., 2025; Candiani et al., 2023; Carlisle et al., 2021; Chen et al., 2024; Contreras et al., 2021; Csiplo & Popescu, 2025; da Silva et al., 2017; de Assis & Mills, 2021; Ellis, 2022; Foreman-Worsley & Farnworth, 2019; Freeman et al., 2016; Fulmer et al., 2022; Jahn & DePorter, 2023; Kogan et al., 2024; Kong et al., 2021; Lamon et al., 2023, 2025, 2026; Loberg & Lundmark, 2016; Lorbach et al., 2025; Marangoni et al., 2023; Marangoni & Steagall, 2024; Mariti et al., 2017; McPeake et al., 2023; Mills et al., 2014; Monteiro et al., 2023; Nagasawa et al., 2022; Nicholson & O'Carroll, 2021; Paz et al., 2022; Pereira et al., 2016; Powell et al., 2023; Rehnberg et al., 2015; Siguín et al., 2025; Steagall et al., 2022; Steagall & Monteiro, 2019; Stella & Croney, 2019; Takagi et al., 2023; Tatlock et al., 2017; Van Der Leij et al., 2019; Vinke et al., 2014; Vojtkovská et al., 2020, 2021; Zito et al., 2019)
	Reviews	7	(Foreman-Worsley & Farnworth, 2019; Fulmer et al., 2022; Jahn & DePorter, 2023; Lamon et al., 2023; Mills et al., 2014; Steagall & Monteiro, 2019; Vojtkovská et al., 2020)
	Scientific report	1	(Candiani et al., 2023)
	Original articles	36	(Bigras-Fontaine et al., 2025; Carlisle et al., 2021; Chen et al., 2024; Contreras et al., 2021; Csiplo & Popescu, 2025; da Silva et al., 2017; de Assis & Mills, 2021; Ellis, 2022; Freeman et al., 2016; Kogan et al., 2024; Kong et al., 2021; Lamon et al., 2025, 2026; Loberg & Lundmark, 2016; Lorbach et al., 2025; Marangoni et al., 2023; Marangoni & Steagall, 2024; Mariti et al., 2017; McPeake et al., 2023; Monteiro et al., 2023; Nagasawa et al., 2022; Nicholson & O'Carroll, 2021; Paz et al., 2022; Pereira et al., 2016; Powell et al., 2023; Rehnberg et al., 2015; Siguín et al., 2025; Steagall et al., 2022; Stella & Croney, 2019; Takagi et al., 2023; Tatlock et al., 2017; Van Der Leij et al., 2019; Vinke et al., 2014; Vojtkovská et al., 2021; Zito et al., 2019)
**Welfare assessment approach (original articles)**	Multiple parameters	20	(Bigras-Fontaine et al., 2025; Carlisle et al., 2021; Chen et al., 2024; Contreras et al., 2021; Ellis, 2022; Kong et al., 2021; Lamon et al., 2026; Loberg & Lundmark, 2016; Lorbach et al., 2025; Nagasawa et al., 2022; Paz et al., 2022; Pereira et al., 2016; Rehnberg et al., 2015; Siguín et al., 2025; Steagall et al., 2022; Stella & Croney, 2019; Takagi et al., 2023; Van Der Leij et al., 2019; Vojtkovská et al., 2021; Zito et al., 2019)

	Different categories	9	(Carlisle et al., 2021; Chen et al., 2024; Contreras et al., 2021; Lorbach et al., 2025; Nagasawa et al., 2022; Paz et al., 2022; Rehnberg et al., 2015; Stella & Croney, 2019; Takagi et al., 2023; Van Der Leij et al., 2019)
**Parameter types used**	Behavioural	30	(Bigras-Fontaine et al., 2025; Carlisle et al., 2021; Chen et al., 2024; Contreras et al., 2021; Csiplo & Popescu, 2025; de Assis & Mills, 2021; Ellis, 2022; Freeman et al., 2016; Kogan et al., 2024; Lamon et al., 2026; Loberg & Lundmark, 2016; Lorbach et al., 2025; Marangoni et al., 2023; Marangoni & Steagall, 2024; Mariti et al., 2017; McPeake et al., 2023; Monteiro et al., 2023; Nagasawa et al., 2022; Nicholson & O'Carroll, 2021; Paz et al., 2022; Pereira et al., 2016; Powell et al., 2023; Rehnberg et al., 2015; Siguín et al., 2025; Steagall et al., 2022; Stella & Croney, 2019; Takagi et al., 2023; Tatlock et al., 2017; Van Der Leij et al., 2019; Vinke et al., 2014)
	Physiological	10	(Carlisle et al., 2021; Chen et al., 2024; Contreras et al., 2021; da Silva et al., 2017; Lamon et al., 2025; Nagasawa et al., 2022; Paz et al., 2022; Rehnberg et al., 2015; Siguín et al., 2025; Stella & Croney, 2019)
	Health-related	7	(Carlisle et al., 2021; Kong et al., 2021; Lorbach et al., 2025; Paz et al., 2022; Van Der Leij et al., 2019; Vojtkovská et al., 2021; Zito et al., 2019)
**Methodological features**	At least one validated parameter used	31	(Bigras-Fontaine et al., 2025; Carlisle et al., 2021; Chen et al., 2024; Contreras et al., 2021; de Assis & Mills, 2021; Foreman-Worsley & Farnworth, 2019; Freeman et al., 2016; Fulmer et al., 2022; Kogan et al., 2024; Lamon et al., 2023, 2025, 2026; Loberg & Lundmark, 2016; Lorbach et al., 2025; Marangoni et al., 2023; Marangoni & Steagall, 2024; McPeake et al., 2023; Monteiro et al., 2023; Nicholson & O'Carroll, 2021; Paz et al., 2022; Pereira et al., 2016; Powell et al., 2023; Rehnberg et al., 2015; Siguín et al., 2025; Steagall et al., 2022; Steagall & Monteiro, 2019; Stella & Croney, 2019; Tatlock et al., 2017; Van Der Leij et al., 2019; Vinke et al., 2014; Vojtkovská et al., 2020)
	No specific context	16	(Contreras et al., 2021; de Assis & Mills, 2021; Freeman et al., 2016; Kogan et al., 2024; Marangoni et al., 2023; Marangoni & Steagall, 2024; Mariti et al., 2017; McPeake et al., 2023; Monteiro et al., 2023; Nagasawa et al., 2022; Nicholson & O'Carroll, 2021; Powell et al., 2023; Rehnberg et al., 2015; Siguín et al., 2025; Takagi et al., 2023; Zito et al., 2019)
	Owner-reported parameters used	15	(Chen et al., 2024; Contreras et al., 2021; Csiplo & Popescu, 2025; de Assis & Mills, 2021; Kogan et al., 2024; Lorbach et al., 2025; Mariti et al., 2017; McPeake et al., 2023; Monteiro et al., 2023; Nagasawa et al., 2022; Pereira et

			al., 2016; Powell et al., 2023; Stella & Croney, 2019; Takagi et al., 2023; Tatlock et al., 2017)
**Most frequently used method**	Cat Stress Score (CSS)	9	(Bigras-Fontaine et al., 2025; Carlisle et al., 2021; Lamon et al., 2026; Loberg & Lundmark, 2016; Paz et al., 2022; Pereira et al., 2016; Rehnberg et al., 2015; Van Der Leij et al., 2019; Vinke et al., 2014)
